# Characterization of a Novel Simian Sapelovirus Isolated from a Cynomolgus Monkey using PLC/PRF/5 Cells

**DOI:** 10.1038/s41598-019-56725-z

**Published:** 2019-12-27

**Authors:** Wenjing Zhang, Michiyo Kataoka, Hai Yen Doan, Yasushi Ami, Yuriko Suzaki, Naokazu Takeda, Masamichi Muramatsu, Tian-Cheng Li

**Affiliations:** 1 Blood Center of Shandong Province, East Shanshi Road 22, Jinan, Shandong 250014 China; 2 Department of Virology II, National Institute of Infectious Diseases, Gakuen 4-7-1, Musashi-murayama, Tokyo 208-0011 Japan; 3 Department of Pathology, National Institute of Infectious Diseases, Gakuen 4-7-1, Musashi-murayama, Tokyo 208-0011 Japan; 4 Division of Experimental Animals Research, National Institute of Infectious Diseases, Gakuen 4-7-1, Musashi-murayama, Tokyo 208-0011 Japan; 5 Research Institute for Microbial Diseases, Osaka University, Suita, Osaka 565-0781 Japan

**Keywords:** Virology, Microbiology, Zoology

## Abstract

We isolated a novel simian sapelovirus (SSV), Cam13, from fecal specimen of a cynomolgus monkey by using PLC/PRF/5 cells. The SSV infection of the cells induced an extensive cytopathic effect. Two types of virus particles with identical diameter (~32 nm) but different densities (1.348 g/cm^3^ and 1.295 g/cm^3^) were observed in the cell culture supernatants. The RNA genome of Cam13 possesses 8,155 nucleotides and a poly(A) tail, and it has a typical sapelovirus genome organization consisting of a 5’ terminal untranslated region, a large open reading frame (ORF), and a 3’ terminal untranslated region. The ORF encodes a single polyprotein that is subsequently processed into a leader protein (L), four structural proteins (VP1, VP2, VP3, and VP4) and seven functional proteins (2A, 2B, 2C, 3A, 3B, 3C, and 3D). We confirmed that 293 T, HepG2/C3A, Hep2C, Huh7 and primary cynomolgus monkey kidney cells were susceptible to SSV infection. In contrast, PK-15, Vero, Vero E6, RD-A, A549, and primary green monkey kidney cells were not susceptible to SSV infection. We established an ELISA for the detection of IgG antibodies against SSV by using the virus particles as the antigen. A total of 327 serum samples from cynomolgus monkeys and 61 serum samples from Japanese monkeys were examined, and the positive rates were 88.4% and 18%, respectively. These results demonstrated that SSV infection occurred frequently in the monkeys. Since Cam13 shared 76.54%–79.52% nucleotide sequence identities with other known SSVs, and constellated in a separate lineage in the phylogeny based on the entire genome sequence, we propose that Cam13 is a new genotype of the simian sapelovirus species.

## Introduction

Many simian viruses (SV) were isolated from various primate tissues during the development of tissue culture methods as well as from specimens derived from primates used in biomedical research^1–7^. Most of these viruses have been classified into the genus *Enterovirus* of the family *Picornaviridae*^8,9^. It has been demonstrated that some SV strains (e.g., SV2, SV16, SV18, SV42, SV44, SV45 and SV49) are distinct from other simian enteroviruses, and rather related to porcine enterovirus 8 (PEV8)^10,11^. Based on the entire genome sequences, SV2 and PEV8 were classified into a new genus *Sapelovirus*^11^, which currently consists of two species: *Sapelovirus A* (formerly known as porcine sapelovirus [PSV]), and *Sapelovirus B* (formerly named as simian sapelovirus [SSV])^12^. A third species (*Avian sapelovirus*) including duck picornavirus (DPV) TW90A has been renamed *Anativirus A* and moved to a new genus, *Anativirus* (http://www.picornaviridae.com/sapelovirus/sapelovirus.htm)^13^.

Sapelovirus is a nonenveloped positive-sense single-stranded RNA viruses. It contains approximately 7.5~ 8.2 kb genomic RNA. The genome organization is similar to that of other picornaviruses: a 5′ untranslated region (UTR), a large open reading frame (ORF), a 3′ UTR, and poly(A) tail. The large ORF encodes a single polyprotein that is subsequently processed into four structural proteins (VP4, VP2, VP3 and VP1) and seven non-structural proteins (2A, 2B, 2C, 3A, 3B, 3C and 3D) (http://www.picornaviridae.com/). In addition, sapeloviruses possess a leader protein (L) at the N-terminus of the polyprotein^10,14^.

PSV has been detected in pigs worldwide, and the entire genome sequences of several PSV strains have been analyzed^15–19^. In contrast, only four entire genome sequences of SSV are available to date. Based on the VP1 amino acid (aa) sequences the SSVs can be separated into three groups (SSV1, SSV2 and SSV3)^11^, but no clear genotyping for these viruses has been reported, and the epidemiology, antigenicity, and pathogenicity of the SSVs remain unclear. In the present study, we used a human hepatocarcinoma cell line, PLC/PRF/5, to isolate an SSV from fecal specimens of cynomolgus monkeys. The next-generation sequence analysis (NGS) of the entire genome of our isolate (Cam13) suggested that this virus is a novel member of SSV. We also proposed the introduction of a genotyping system to classify SSV isolates among *Sepelovirus B*.

## Materials and Methods

### Fecal and serum samples from monkeys

A set of serum and fecal samples was collected from 30 cynomolgus monkeys (C1 to C30) originally to detect hepatitis E virus (HEV)^20^. The monkeys were 3–4 years old and imported from a monkey farm in Cambodia in 2017. We also used a total of 297 serum samples including 36 sera collected from cynomolgus monkeys bred and grown at the Tsukuba Primate Research Center, Japan in 1995; 123 serum samples from cynomolgus monkeys imported from China in 2017; 58 serum samples from wild cynomolgus monkeys imported from the Philippines in 1985; and 80 sera from wild cynomolgus monkeys imported from Malaysia in 1969.

All of the serum samples from the imported monkeys were collected right after the monkeys were imported into Japan. In addition to cynomolgus monkeys, 61 serum samples collected from Japanese monkey from 1995 to 2004 in Japan were used. The serum samples and stool suspensions were stored at −80 °C until use. The imported monkeys used in these experiments were reviewed and approved by the institutional ethics committee of the National Institute of Infectious Diseases (NIID), and all of the animal experiments were carried out according to the “Guides for Animal Experiments Performed at NIID”.

### Cell culture and virus inoculation

The cell line PLC/PRF/5 (JCRB0406) was obtained from the Health Science Research Resources Bank (Osaka, Japan). The cells were grown in Dulbecco’s modified Eagle’s medium (DMEM) with L-glutamine and high glucose supplemented with 10% (v/v) heat-inactivated fetal bovine serum (FBS; Nichirei, Biosciences, Tokyo), 100 U penicillin (Gibco, Grand Island, NY) and 100 mg streptomycin (Gibco) at 37 °C in a humidified 5% CO_2_ atmosphere. For virus inoculation, the confluent cells were trypsinized, diluted 1:3 and cultured in a 25-cm^2^ tissue culture flask. After 24 h of incubation, the medium was removed and the cells were washed two times with phosphate-buffered saline (PBS)^21^.

Ten fecal specimens from monkeys that were positive for anti-HEV IgM antibody by an enzyme-linked immunosorbent assay (ELISA) were selected to inoculate PLC/PRF/5^20^. The fecal specimens were diluted with 10 mM PBS to prepare a 10% (w/v) stool suspension. The suspension was shaken at 4 °C for 1 h, clarified by centrifugation at 10,000 *g* for 30 min, and passed through a 0.45 µm membrane filter (Millipore, Bedford, MA)^22^.

One ml of the 10% stool suspension was inoculated onto PLC/PRF/5 cells, and the adsorption was performed at 37 °C for 1 h. The cells were washed two times with PBS and then supplemented with 10 ml of maintenance medium consisting of medium 199 (Invitrogen, Carlsbad, CA) containing 2% (v/v) heat-inactivated FBS and 10 mM MgCl_2_. Further incubation was done at 36 °C. The culture medium was collected and supplemented with new medium every 4 days^22^.

Two green monkey kidney cell lines (Vero and Vero E6), green monkey primary kidney cells (PGMKC), cynomolgus monkey primary kidney cells (PCMKC), a human embryonic kidney cell line (293 T), a porcine kidney cell line (PK-15), human hepatocellular carcinoma cell lines (HepG3/C3A, Huh-7.5.1), a human lung carcinoma cell line (A549), a human cervix adenocarcinoma cell line (Hep2c), and an embryonal rhabdomyosarcoma cell line (RD-A) were used to examine the susceptibility to SSV. These cells were inoculated with the supernatant of SSV-infected PLC/PRF/5 cells. All of the cells were cultured with DMEM supplemented with 10% FBS at 37 °C in a humidified 5% CO_2_ atmosphere.

### Purification of virus particles

The virus–infected PLC/PRF/5 cells were harvested when CPE completely appeared and then centrifuged at 10,000 *g* for 60 min to remove the cells and the debris. The supernatant was then centrifuged at 32,000 rpm for 3 h in a Beckman SW32Ti rotor. The resulting pellet was re-suspended in PBS buffer at 4 °C overnight, and purified by an equilibrium CsCl gradient centrifugation at 35,000 rpm for 24 h at 10 °C in a Beckman SW55Ti rotor. The gradient was fractionated into 250 μl aliquots, and each fraction was weighed to estimate the buoyant density and isopycnic point. Finally, each fraction was diluted with PBS and centrifuged for 2 h at 50,000 rpm in a Beckman TLA55 rotor to remove CsCl^16^.

### SDS-PAGE and western blotting

The viral proteins in each fraction were separated by SDS-PAGE using 5%–20% e-Pagel (ATTO, Tokyo) and then stained with Coomassie blue. For Western blotting, the separated proteins were electrophoretically transferred onto a nitrocellulose membrane. The membrane was blocked with 5% skim milk in 50 mM Tris-HCl (pH 7.4) containing 150 mM NaCl, and then incubated with 1:500 diluted monkey serum. Detection of the monkey IgG antibody was achieved using phosphatase-labeled goat anti-monkey IgG (H + L) (1:1000 dilution) (abcam, Tokyo). Nitroblue tetrazolium chloride and 5-bromo-4-chloro-3-indolyl phosphate P-toluidine were used as coloring agents (Bio-Rad Laboratories, Hercules, CA)^16^.

### Electron microscopy (EM)

The purified virus particles were placed on a carbon-coated grid for 45 sec, rinsed with distilled water, and stained with a 2% uranyl acetate solution. The grids were observed under a transmission electron microscope (HT7700; Hitachi High Technologies, Tokyo, Japan) at 80 kV^16,23^.

### Viral genome sequencing

The RNA was extracted from the purified virus particles by using a MagNA Pure LC system with a MagNA Pure LC Total Nucleic Acid isolation kit (Roche Applied Science, Mannheim, Germany) according to the manufacturer’s recommendations. The nucleotide sequences of the entire genomes were determined by NGS as described previously^24^. The 3′-terminus nucleotide sequence was further confirmed by RT-PCR. The reverse transcription was performed by using Superscript^™^ II RNase H^−^ reverse transcriptase (Invitrogen, Carlsbad, CA) and oligonucleotide (dT) primer TX30SXN (5′-GACTAGTTCTAGATCGCGAGCGGCCGCCCTTTTTTTTTTTTTTTTTTTTTTTTTTTTTT-3′)^25^. The 3′-terminal sequence was amplified by PCR using the primers, camF7879 (5′-ATGGACACGTAGCGCTGCTA-3′) and TX30SXN. The nucleotide sequencing was carried out with the primer camF7879 using an ABI 3130 Genetic Analyzer automated sequencer (Applied Biosystems, Foster City, CA).

### ELISA to detect anti-SSV IgG antibodies

In reference to the detection of the anti-HEV IgG antibodies, we established an ELISA for the detection of anti-SSV IgG antibodies by using SSV particles as the antigen^26^. Briefly, 96-well polystyrene microplates (Immulon 2; Dynex Technologies, Chantilly, VA) were coated with the purified empty particles of Cam13 (1 µg/mL, 100 µl/well) and incubated overnight at 4 °C. The plates were washed twice with 10 mM PBS containing 0.05% Tween 20 (PBS-T), blocked with 200 µl of 5% skimmed milk (Difco, Sparks, MD) dissolved in PBS-T for 1 h at 37 °C, and washed three times as described above. Next, 100 µl of the diluted serum samples (1:200 dilutions) was added and incubated at 37 °C for 1 h.

After three consecutive washes, each well was supplemented with 100 µl of horseradish peroxidase (HRP)-conjugated goat anti-monkey IgG-heavy and light chain antibody (Bethyl Laboratories, Montgomery, TX) (1:10,000 dilution). The plates were incubated at 37 °C for 1 h and then were washed four times with PBS-T. The substrate orthophenylenediamine (100 µl) (Sigma, St. Louis, MO) supplemented with H_2_O_2_ was added to each well. The plates were incubated in a dark room at room temperature for 30 min, and then 50 µl of 2 M H_2_SO_4_ was added to each well. Optical density (OD) values were measured at 492 nm.

### Phylogenetic analyses

The nucleotide (nt) and amino acid (aa) sequences were aligned in the ClustalW software followed by phylogenetic analysis via the maximum-likelihood method in the MEGA 6 software. The phylogenetic trees were evaluated by bootstrap analysis with 1000 replicates.

## Results

### Replication of cytopathic effect (CPE) agent(s) in PLC/PRF/5 cells

Monkey fecal specimens were originally used to isolate HEV, as described previously^20^. However, when we used the sterile-filtered 10% stool suspensions to inoculate PLC/PRF/5 cells, an unexpected CPE was observed on day 7 post-inoculation (p.i.) in the cells that received the suspension from 1 of 10 monkeys imported from Cambodia (C13), although no HEV RNA was detected in the supernatant. We thus collected the cell culture supernatant and used it again to inoculate PLC/PRF/5 cells. The CPE was clearly observed at day 2 p.i., and again no HEV RNA was detected in the supernatant, indicating that the CPE was unlikely to be caused by HEV infection.

**Figure 1:**
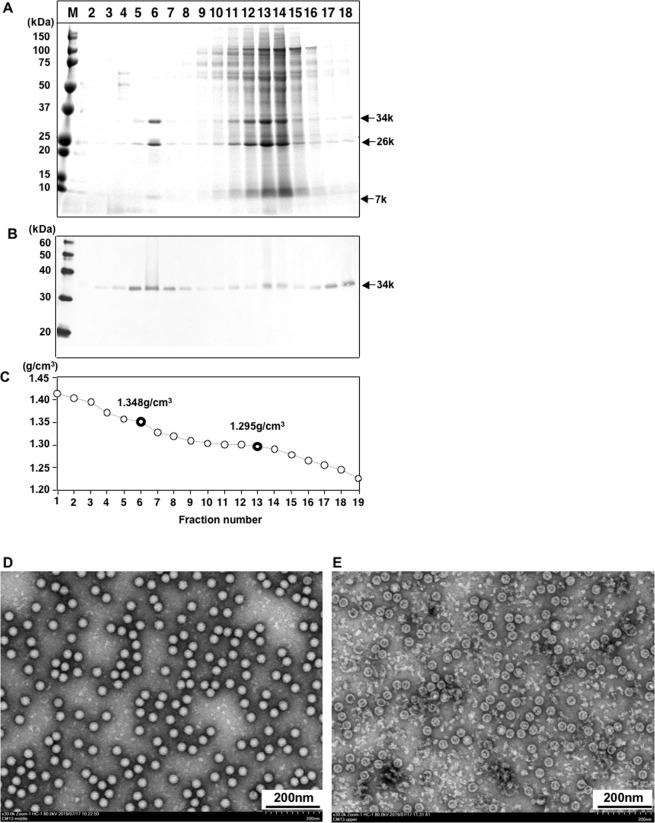
Purification of CPE agent (s). The supernatant of the infected PLC/PRF/5 cells was concentrated by ultracentrifugation, and further purified by CsCl gradient centrifugation. Aliquots from each fraction were analyzed by 5%–20% SDS-PAGE. Proteins were visualized by Coomassie blue staining (**A**) and western blotting with SSV-positive serum from Monkey C13 (**B**). The density of each fraction is shown (**C**). Virus particles in fraction 6 (**D**) and 13 (**E**) were observed by EM. Bar, 200 nm.

To purify the CPE agent(s), we harvested the infected PLC/PRF/5 cells at day 4 p.i.; the supernatant was concentrated, and the pelletized agent(s) was subjected to equilibrium CsCl density gradient ultracentrifugation. The gradient was separated into 20 fractions and each fraction was examined by SDS-PAGE. The protein bands were primarily distributed in fractions 6 and 11–14 with densities of 1.348 and 1.295 g/cm^3^, respectively (Fig. 1A,C).

In fraction 6, at least three protein bands were observed with molecular masses of ~34 kDa, ~26 kDa and ~7 kDa, whereas two protein bands with molecular masses of ~34 kDa, and ~26 kDa were observed in fractions 11–14 (Fig. 1A). By western blotting, the 34 kDa protein reacted with the serum that was collected from monkey C13 (Fig. 1B). Electron microscopy (EM) of fraction 6 showed many spherical virus particles with a diameter of approximately 32 nm (Fig. 1D), and the morphology of these particles was similar to that of PSV as observed in our previous studies. In contrast, only empty particles with the same diameter were observed in fractions 11–14 (Fig. 1E).

### The entire genome sequences of CPE agents

**Figure 2:**
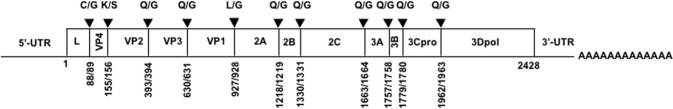
Genome organization of the SSV Cam13 strain. The genome organization and aa sequences of the putative cleavage sites of the polyprotein of Cam13 are indicated.

For our analysis of the entire genome sequences of the CPE agent (s), the RNA was extracted from the purified particles in fraction 6 and subjected to NGS analyses. The sequence analyses revealed that the genomes consisted of 8,155 nucleotides (nt) and a poly (A) tail (GenBank accession no. LC503602). The 5′-terminal untranslated region (5′UTR) and the 3′UTR contained 773 and 95 nucleotides, respectively, and the strains contained a single large ORF with 7,287 nt encoding 2,428 aa. The predicted protease cleavage sites Q/G were found in the aa positions 393/394, 630/631, 927/928, 1,218/1,219, 1,330/1,331, 1,663/1,664, 1,757/1,758, 1,779/1,780 and 1,962/1,963 of the polyprotein (Fig. 2).

We also observed putative cleavage sites, C/G in aa positions 88/89, K/S in aa positions 155/156 and L/G in aa positions 927/928. Based on these cleavage sites and the genome organization, this CPE agent was similar to known sapelovirus strains, and the polyprotein seemed to be cleaved into twelve mature peptides consisting of an L protein, the four structural proteins VP4, VP2, VP3 and VP1, and the seven non-structural proteins 2A, 2B, 2C, 3A, 3B, 3C and 3D (Fig. 2). The results of our comparison of the predicted SSV and PSV and TW90A proteins are summarized in Table 1. A BLAST analysis showed that the nucleotide sequence was mostly similar to that of SSV, and the genome shared 76.54–79.52% nucleotide sequence identities with known SSVs. These results demonstrated that the CPE agent in the cynomolgus monkey fecal specimens is a SSV and we designated this strain as Cam13.

**Table 1:** Comparison of proteins and cleavage sites of sapeloviruses and avian anativirus TW90A.

Proteins	Predicated protein size, aa				Cleavage sites			
	^a^Cam13	^b^SV2	^c^HEV8	^d^TW90A	Cam13	SV2	PSV	TW90A
Polyprotein	2428	2429	2180	2521	—	—	—	—
L	88	88	84	451	—	—	—	—
VP4	67	67	53	69	C/G	C/G	Q/G	Q/G
VP2	238	238	238	253	K/S	K/K	K/A	Q/N
VP3	237	241	234	232	Q/G	Q/G	Q/G	Q/G
VP1	297	284	285	290	Q/G	Q/G	Q/G	Q/G
2 A	291	302	226	12	L/G	T/G	L/G	T/V
2B	112	112	105	108	Q/G	Q/G	Q/G	Q/G
2 C	333	333	332	333	Q/G	Q/G	Q/G	Q/G
3 A	94	94	100	103	Q/G	Q/G	Q/G	Q/G
3B	22	22	22	22	Q/G	Q/G	Q/G	Q/G
3 C	183	182	182	185	Q/G	Q/G	Q/G	Q/G
3D	466	466	461	163	Q/G	Q/G	Q/G	Q/G

^a^LC503602, SSV^b^. AY064708, SSV^c^. AF406813, PSV^d^. AY563023.

**Figure 3:**
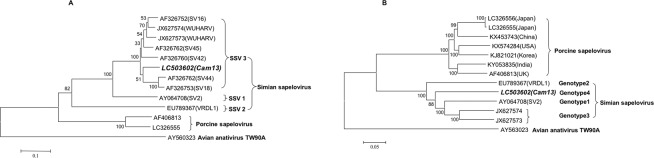
Phylogenetic relationships among SSV strains. The amino acid and nucleotide sequence alignment was performed using ClustalW software, with an Avian anativirus TW90A as the out-group. The genetic distance was calculated by Kimura’s two-parameter method. Phylogenetic trees with 1,000 bootstrap replicates were generated by the Neighbor-Joining method using MEGA (ver. 6) based on the aa sequence of VP1 (**A**) and the entire genome (**B**) of SSV, PSV, and TW90A. Scale bar indicates amino acid or nucleotide substitutions per site. Strain Cam13 strain is shown in *bold italic* letters. The genotype is tentatively named based on the findings of our present study.

Although many SSV strains including of SV4, SV16, SV18, SV42, SV45 and SV49, have been isolated from monkey kidney cells, only partial sequences were analyzed^1–3^. The phylogenetic analyses based on the aa sequences of VP1 indicated that SSV separated into three types, SSV1, SSV2, and SSV3, and Cam13 belongs to SSV3 (Fig. 3A). However, the phylogenetic analysis based on the entire genome of the SSV strains clearly indicated that Cam13 is a member of SSV but belongs to a separate cluster (Fig. 3B). Of the five entire genome of SSV strain, those of two strains (JX627573 and JX 627574)^27^ collected from rhesus monkeys were the most closely related, and the nucleotide sequence identity between these two strains was 87.36%. These two strains shared 82.10–82.86% and 82.30–83.69% of the nucleotide identities with the VRDL1 strain (EU789367)^28^ and SV2 strain (AY064708)^11^, respectively. In contrast, Cam13 shared 76.58%, 78.12%, 79.21% and 79.52 of the nucleotide sequence identity with VRDL1, SV2, JX627573 and JX627574, respectively. In addition, the VP1 of Cam13 shared 60.00%, 70.25%, 76.11% and 76.83% of the nucleotide sequence identity with that of VRDL1, SV2, JX627573 and JX627574, respectively (Tables 2).

**Table 2:** Nucleotide sequence identities (%) among VP1 of SSV strains.

SSV strains	LC503602 (Cam13)	JX627573	JX627574	AY064708 (SV2)
JX627573	76.11			
JX627574	76.83	83.83		
AY064708 (SV2)	70.25	70.38	69.42	
EU789367 (VRDL1)	60.00	61.00	58.48	59.54

**Table 3:** Susceptibility of the cell lines to SSV and PSV.

Cell line	SSV (Cam13)	PSV (Jpsv1315)
PLC/PRF/5	+	+
293 T	+	+
HepG2/C3/A	+	+
PCMKC	+	−
Hep2C	+	−
Huh7.5.1	+	−
PK-15	−	+
PGMKC	−	+
VeroE6	−	+
RD-A	−	+
Vero	−	−
A549	−	−

+CPE was observed. −No CPE was observed.

Based on these results, we propose that SSV species can be segregated into four genotypes: the first isolate SV2 belongs to genotype 1, JX627573 and JX 627574 belong to genotype 2, and VRDL1 belongs to genotype 3 according to the time of their isolation. Therefore, Cam13 should be classified into genotype 4 (Fig. 3).

### Susceptibility of other cell lines to SSV

In our previous research we confirmed that PSV is capable of infecting not only PLC/PRF/5 cells but also PK-15, Vero E6, and PGMKC cells^16^. Here to investigate whether other cell lines are susceptible to SSV, we used Cam13 to infect several cell lines, and we compared the susceptibility with PSV strain, Jpsv1315 (LC32655). As shown in Table 3, both Cam13 and Jpsv1315 induced CPE in PLC/PRF/5, 293 T, HepG2/C3A cells, but not in Vero or A549 cells. Interesting, the difference in susceptibility between SSV and PSV are as the follows: PCMKC, Hep2C and Huh-7.5.1 cells are susceptible to the SSV (Cam13) but not to the PSV (Jpsv1315). In contrast, PK-15, PGMKC, Vero E6 and RD-A cells are susceptible to the PSV (Jpsv1315) but not the SSV (Cam13). These results suggested that PSV and SSV might use different pathway to enter cells.

### Detection of anti-SSV IgG antibodies in monkey serum

**Figure 4:**
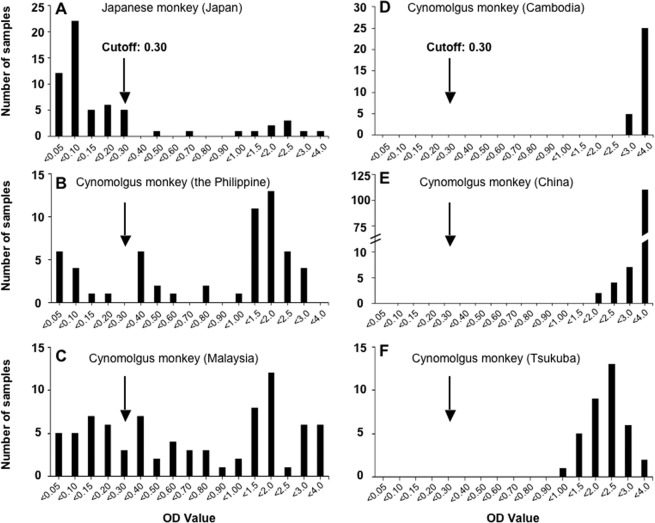
Detection of anti-SSV antibodies in monkeys. Anti-SSV IgG antibodies in 6 groups of monkeys were examined by ELISA using the purified Cam13 virus particles as the antigen. The number of samples for each OD value are plotted. The cutoff value is indicated by an *arrow*.

We used purified Cam13 particles from fraction 6 (Fig. 1) to establish an ELISA for the detection of anti-SSV IgG antibodies. The distribution of the OD values of the antibodies in the serum samples from 388 cynomolgus monkeys is illustrated in Fig. 4. The OD values of the sera from the Japanese monkeys ranged from 0.018 to 3.193, and we observed two peaks from 0.018 to 0.271 and 0.490 to 3.193 (Fig. 4A). When 50 samples with lower OD values were used to determine the cutoff for the ELISA, the mean OD value was 0.099 with a standard deviation (SD) of 0.067, and then the cutoff was calculated as 0.300 on the basis of the mean OD plus 3 times the SD (0.099  + 3 × 0.067).

Since the OD values of the serum samples of the cynomolgus monkeys from Tsukuba, Cambodia, and China ranged from 1.098 to 3.134, 2.610 to 3.537, and 1.504 to 3.564, respectively (Fig. 4D–F), all of these monkeys were positive for anti-SSV IgG. The positive rates of anti-SSV IgG in the wild cynomolgus monkeys imported from the Philippines and Malaysia were 79.3% (46/58) and 67.5% (54/80), respectively (Fig. 4B,C). The anti-SSV IgG positive rate in the whole series of cynomolgus monkeys was 88% (289/327). In contrast, the positive rate of the Japanese monkeys was 18.0% (11/61). These results suggested that (1) SSV infection is common in cynomolgus monkeys and the (2) Japanese monkeys were similarly exposed to SSV infection although the positive rate among them was lower than that in the cynomolgus monkeys.

### Cross-reaction between PSV and SSV

**Figure 5:**
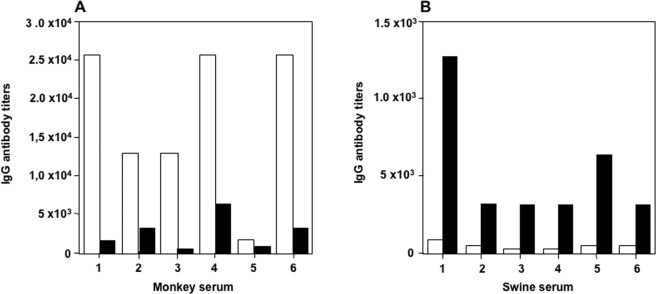
Antigenic cross-reactivity between SSV and PSV. Anti-SSV IgG (white bars) and anti-RSV (black bars) antibody titers in the serum samples from cynomolgus monkeys (**A**) and in the serum sample from swine (**B**) were detected by antibody ELISA.

We examined the antigenic cross-reactivity between SSV and PSV by performing the ELISA. We used the purified virus particles of SSV (Cam13) and PSV (Jpsv1315) to detect the IgG antibodies. Six anti-SSV IgG-positive serum samples from cynomolgus monkeys and six anti-PSV IgG-positive serum samples from swine were used. As shown in Fig. 5, the IgG antibody titers in the monkey serum detected by SSV were higher than those detected by PSV (Fig. 5A). In contrast, the antibody titers in the swine serum detected by PSV were higher than those detected by SSV (Fig. 5B). In addition, anti-SSV IgG-negative monkey serum samples did not react with PSV (Jpsv1315) and anti-PSV IgG negative swine sera did not react with SSV (Cam13) (data not shown). These results indicated that a weak antigenic cross-reaction existed between the PSV and the SSV. Further studies are required to examine whether the anti-SSV IgG neutralizes PSV infection or anti-PSV IgG neutralizes the SSV infection.

## Discussion

To data, several nucleotide sequences including a partial or entire SSV genome have been analyzed, but the epidemiology, biology, and pathology of this virus has remained unclear. In this study we isolated a novel SSV strain, Cam13, from fecal specimens of a cynomolgus monkey by cell culture with PLC/PRF/5 cells and we analyzed physicochemical and immunological characteristics of this virus.

Based on the results of sequence analyses, four capsid proteins (VP1, VP2, VP3, and VP4) were assumed, but only three protein bands were observed by SDS-PAGE from purified particles (Fig. 1A). This is because the genome of VP2 and VP3 encodes 238 aa and 237 aa, and since both proteins have the same molecular mass (26 kDa), they appeared as a single band on the SDS-PAGE. Although, the SSV consists of four capsid proteins, only VP1 was shown to react with SSV-positive monkey serum by Western blotting, suggesting that VP1 contains major antigenic epitopes.

The preparation of the purified Cam13 allowed us to establish an ELISA for the detection of anti-SSV IgG antibody in Japanese monkeys and cynomolgus monkeys from China, Cambodia, Malaysia and the Philippines. The sero-prevalence of SSV was as high as 88.4% (289/327) in the cynomolgus monkeys and 18% (11/61) in Japanese monkeys. These results suggested that (1) all of the monkeys from monkey farms or monkey facilities were exposed to SSV, and (2) SSV infection is common in cynomolgus and Japanese monkeys. In addition, the positive rates of anti-SSV IgG in the wild monkeys were 79.3% (Philippines) and 67.5% (Malaysia), which is significant lower than the rate in the monkeys from monkey farm or facility in Cambodia, China and Tsukuba, Japan. These results indicated that SSV infection in monkeys seems to spread more easily among farms than in wild environment.

Cam13 was isolated from a healthy cynomolgus monkey, and although most of the monkeys described herein have been exposed to SSV, none of the monkeys showed significant clinical signs, suggesting that SSV infection does not cause any serious diseases in monkeys.

As the receptor molecule(s) of SSV is unknown, we compared the susceptibilities of several cell lines to sapeloviruses to obtain clues. We observed that PCMKC, Hep2C, Huh-7.5.1, PK-15, Vero E6, PGMKC, and RD-A cells showed different susceptibilities to PSV and SSV, suggesting that these viruses use different receptors for infection.
